# FCTC ratification, smoking prevalence, and GDP per capita: lessons for Indonesia and the rest of the world

**DOI:** 10.1186/s12992-022-00810-y

**Published:** 2022-02-05

**Authors:** Abdillah Ahsan, Rifai Afin, Nadira Amalia, Martha Hindriyani, Ardhini Risfa Jacinda, Elisabeth Kramer

**Affiliations:** 1 Department of Economics, Faculty of Economics and Business, University of Indonesia, Depok, West Java 16424 Indonesia; 2 Department of Economics, University of Trunojoyo Madura, Banegkalan, Indonesia; 3 Department of Economics, Faculty of Economics and Administration, University of Malaya, Kuala Lumpur, Malaysia; 4 Faculty of Economics and Business, Gadjah Mada University, Yogyakarta, Indonesia; 5 Center for Islamic Economics and Business, Faculty of Economics and Business, University of Indonesia, Depok, Indonesia; 6 Sydney Southeast Asia Centre, University of Sydney, Sydney, Australia

**Keywords:** Framework convention on tobacco control (FCTC), Indonesia, GDP per capita

## Abstract

**Background:**

Indonesia’s stagnated progress towards tobacco control could be addressed through the implementation of a comprehensive national framework, such as the World Health Organization’s (WHO) Framework Convention of Tobacco Control (FCTC). However, national tobacco industry supporters argue that accepting the FCTC will have negative economic implications for the country. These arguments have, thus far, discouraged the Indonesian government from ratifying the FCTC. Drawing from an analysis of the impact of the FCTC on other countries’ smoking rates and Gross Domestic Product (GDP) per capita, this study offers empirical evidence against industry arguments concerning the potential negative economic impacts of FCTC adoption. This study applies a two stage least square estimation strategy to unbalanced panel data at country level. In the first stage we estimate the impact of FCTC ratification on smoking rates, and in the second step, we estimate the influence of smoking activity on macroeconomic performance.

**Results:**

The result of this study shows that FCTC ratification has a negative impact on a country’s smoking prevalence. While FCTC ratification positively correlates with reduced smoking prevalence, a decline in smoking prevalence is not related to a decline in GDP per capita.

**Conclusions:**

The results of this study shows that FCTC ratification, which can be an important driver for more effective tobacco control, does not necessarily have a negative impact on the economy. Instead, FCTC ratification may be beneficial for both health and economic outcomes, as it provides comprehensive guidance for reducing smoking prevalence that take into account social and economic factors.

## Background

In Indonesia, tobacco control indicators over the past decade show stagnated, or even declining, progress. According to data from the Basic Health Research (*Riset Kesehatan Dasar*/RISKESDAS) survey, conducted by the Ministry of Health, there was a slight decrease in smoking prevalence from 2013 to 2018, with overall smoking prevalence going from 29.3 to 28.8% for the male population aged 10+ years. Moreover, there has been a concerning increase in smoking prevalence among females and a consistent rise in smoking prevalence amongst minors aged 10–18 year [1]. Female smoking prevalence nearly doubled from 2.5% in 2013 to 4.8% in 2018, whilst prevalence amongst those aged 10–18 years rose from 7.2% in 2013 to 8.8 and 9.1% in 2016 and 2018 respectively.

**Fig. 1:**
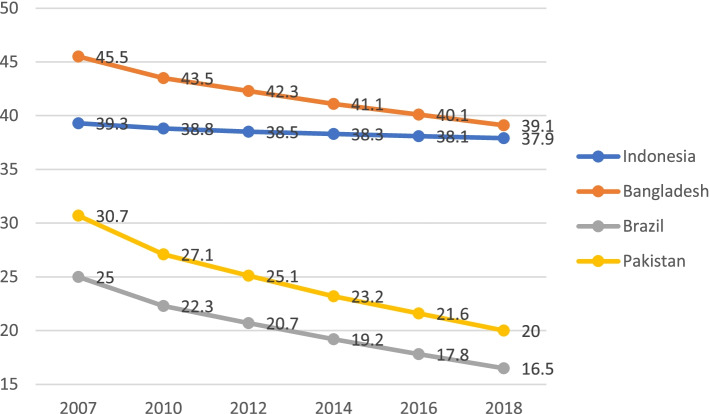
Bangladesh, Brazil, Indonesia, and Pakistan Smoking Prevalence Comparisons 2007–2018 (Age 15+, Both Sexes, Excludes Smokeless Tobacco). Source: World Bank, 2021 [2]

The sub-optimal tobacco control effort in Indonesia is compounded by the lack of a national response framework. The WHO Framework Convention on Tobacco Control (FCTC) is an international protocol that offers a framework for such a response. Indonesia is currently the only country in the Asia-Pacific that has not ratified the FCTC. The FCTC, which was agreed upon by 192 WHO member countries in 2003 and came into effect in 2005, is seen as an important protocol to enforce tobacco control regulation. Comparing Indonesia to other countries with comparable economies that have ratified the FCTC, we can see a difference in smoking prevalence emerged after the FCTC came into effect (Fig. 1).

Figure 1 compares shifts in smoking prevalence between Bangladesh, Brazil, Pakistan, and Indonesia. These four countries have a relatively large number population and are similarly classified as middle-income developing countries [3], with all but Indonesia having yet ratified the FCTC. Bangladesh ratified the FCTC in 2004, while Pakistan and Brazil ratified it in 2005. Figure 1 demonstrates that overall smoking rates have consistently decreased in these three countries since 2007. Whereas over the same period, Indonesia’s smoking prevalence only decreased by 1.4%, a slightly higher figure as compared to RISKESDAS data of 0.4% [4]. This difference is possible due to different age groups used in both dataset. Whereas World Bank data above shows the prevalence among population of age 15+, RISKESDAS data used the prevalence among population of age 10+. Brazil, being the world’s largest tobacco exporter, has successfully decreased its smoking prevalence rate to 16.5%, which is below the global smoking prevalence rate of 23.8%. Among the four countries, Brazil is the only country to have enacted all of the MPOWER^1^ measures, which offer implementation guidance for articles of the FCTC, including a remarkably high tobacco tax of 83% [5]. While Pakistan and Bangladesh have not implemented all MPOWER measures, both countries have addressed the important policy requirements of the FCTC [6, 7]. In comparison, Indonesia has registered a steady increase in smoking prevalence. Being neither a party nor a signatory of FCTC, and with very weak policies on tobacco advertising bans and cigarette affordability, this is no surprise [8, 9].

Economic concerns are the largest political barrier to Indonesia’s ratification of the FCTC. Strong political discourse exists that FCTC ratification will negatively impact the economy through the decline in tobacco production and economic activities associated with the national tobacco industry [10]. This argument holds that stricter tobacco control measures will constrain the industry, leading to a decline in tobacco production that will have a follow-on effect for tobacco farmers’ welfare as well as unemployment for tobacco industry workers. This confluence will, in turn, lead to major macroeconomic problems including an increase in the poverty and unemployment rates and a decrease in GDP per capita. Despite the strong political support for this view, there is no evidence to support this argument. The findings of this paper offer a rebuttal to these arguments by comparing the economic conditions of countries that have ratified the FCTC since 2005 and analysing whether signing the FCTC has any correlation with a decline in GDP per capita in other countries.

## Methods

The study employs unbalanced panel data using a two-stage least square (2SLS) estimation analysis. The 2SLS regression technique involves two stages. In the first stage, we estimated the impact of FCTC ratification on smoking prevalence in signatory countries. In the second stage, the instrument-estimated value from stage one was then computed as a predictor to estimate the impact of smoking activity on the economy. The sample in the panel dataset consists of 87 countries. Due to data availability, the economic activities were traced over the period of 2010–2016. As the focus on this research is to analyse the impact of FCTC ratification at the country level, the primary sample is comprised of countries that ratified the FCTC since 2005.

The model to be estimated is given by

1 $${Y}_{i,t}={\beta}_0+{\upbeta}_i{X}_{i,t}+{\upbeta}_i{T}_{i,t}+{\varepsilon}_{i,t}$$

In which a *Y* is the dependent variable of smoking prevalence, *X* are the independent factors that explain the smoking behaviour, *T* is a variable of time counts of ratification or the length or the duration of a country’s FCTC ratification in year since 2005. Subscripts *i* and *t* explains correspondences of country *i* at time *t*, whereas *ε* is an error term. Variables and sources of data used in this study is outlined in Table 1.

**Table 1:** List of variables

Variables	Description	Source
Smoking Prevalence	Percentage of male smoking prevalence based on males who currently smoke any tobacco product	World Bank
Excise Tax	Percentage of cigarette excise taxes	WHO
Productive Age	Percentage working-age population out of total population. The working-age is defined as those aged 15 to 64	World Bank
Mean Years of Schooling	Average years of education amongst population	Our World in Data
Exchange Rate	Exchange rate of each country to the US dollar	World Bank
Consumer Price Index	Changes in the price level of goods and services purchased by consumers	World Bank
Value-Added Agriculture	Value-added agriculture calculated as a percent of GDP	World Bank
Democratization Index	Index scoring to measure the country democracy	Economist Intelligence Unit
Corruption Perceptions Index	Index scoring based on perceptions of government corruption	Transparency International
Openness	The difference between total exports and imports of goods and services measured as a share of GDP	World Bank
Time count of ratification	Duration since country ratified of FCTC (in years)	WHO

Source: Author’s definition

After regressing the first model (model (1)), with the hypothesis of existing correlation between FCTC ratification and smoking behaviour, the effect of FCTC ratification was then linked to relevant macroeconomic indicators. In the second model estimation (model (2)), the macroeconomic proxy used as a dependent variable is per capita GDP. The effect of FCTC ratification is denoted as $\hat{\ Y}$, smoking prevalence hat (the difference between actual smoking prevalence and the first regression residual value). M denotes other macroeconomic variables representing correlation between macroeconomic indicators and per capita GDP. Subscripts *i* and *t* explains correspondences between country *i* at time *t*, whereas *ε* is an error term.

2 $${Z}_{i,t}={\beta}_0+{\uptheta}_i{\hat{Y}}_{i,t}+{\upbeta}_i{M}_{i,t}+{\varepsilon}_{i,t}$$

## Results

Table 2 summarizes the descriptive statistics of the unbalanced panel datasets. In case some data are missing and not available in particular countries and years, the countries included in the sample are those that have the availability of smoking prevalence dataset.

**Table 2:** Descriptive statistics

Variable	Mean	Std. Dev.	N
Smoking Prevalence	35.217	13.271	609
Excise Tax	0.203	0.173	595
Productive Age	63.867	6.812	602
Mean Years of Schooling	8.429	3.164	567
Exchange Rate	723.222	2664.073	554
Consumer Price Index	114.443	20.012	581
Value Added Agriculture	11.097	11.100	594
Democratization Index	5.522	2.006	564
Corruption Index	43.843	16.803	402
Openness	0.015	0.064	545
Time count of ratification	35.217	13.271	609

Source: Authors calculation

The dependent variables in eq. (1) describes the male smoking prevalence percentage per total male adult population (see Table 3). Our variable of interest in FCTC ratification is the duration of FCTC ratification (measured in years). Control variables that describe smoking behaviour include excise tax as a variable (percentage of cigarette excise taxes) and the productive age of the population.

**Table 3:** Regression estimation of Eq. 1

Dependent: Smoking Prevalence	
Time Count of Ratification	−0.362***
	(0.03)
Excise Tax	−4.835***
	(1.23)
Productive Age	0.053**
	(0.10)
Constant	35.212*
	(6.583)
N observations	588
N-degree of freedom	501
BIC	2102.963
R-squared	0.2665

Note: The dependent variable is the male smoking prevalence of male overall adults. Standard errors are shown in parentheses. ***, ** and * denote *p*-value < 0.01, < 0.05 and < 0.10 respectively.

Source: Authors calculation

The FCTC coefficient (duration of ratification) appears to significantly affect smoking prevalence. This implies that the longer a country has ratified the FCTC, the lower the smoking prevalence will be. Furthermore, the percentage of cigarette taxes is also statistically significant to the smoking prevalence. This means that tax on cigarette affects smoking behaviour, and increasing taxes tend to drive people to reduce smoking. However, where there is a higher percentage of the population within a productive age there shows an insignificant positive relationship to smoking prevalence. This means that the smoking behaviour in a country is not driven by total production age in isolation.

The results from undertaking Eq. 2 show an insignificant relationship between smoking behaviour and per capita GDP (see Table 4). Intriguingly, the smoking prevalence hat shows an inverse relationship with per capita GDP. This indicates that the higher the smoking prevalence, the lower per capita GDP will be. Unfortunately, the relationship between the two variables is not statistically significant.

**Table 4:** Regression estimation of Eq. 2

Dependent: GDP per capita (log)	
Smoking Prevalence hat	−.001
	(0.00)
Mean years of schooling	0.092***
	(0.01)
Exchange rate (log)	0.082**
	(0.03)
CPI (log)	−0.151**
	(0.05)
Value added Agriculture (%)	−0.008***
	(0.00)
Democratization Index	0.061***
	(0.01)
Openness	−0.093
	(0.01)
Corruption Index	0.004***
	(0.00)
Constant	7.85***
	(0.24)
N observations	311
N-degree of freedom	235
BIC	− 1214.661
R-squared	0.3939

Standard errors are shown in parentheses. ***, ** and * denote *p*-value < 0.01, < 0.05 and < 0.10 respectively.

Source: Authors calculation

Based on the results of the regression estimation, if a country has ratified the FCTC, the country will experience a decline in smoking prevalence. However, the changes in smoking prevalence are not statistically significant when compared to other macroeconomic indicators. The results suggest that that being a party to the FCTC will not directly impact a country’s macroeconomic indicators.

## Discussion

The results above imply two factors that are of interest to this study. Firstly, this study found a negative association between the number of years since FCTC ratification and changes in smoking prevalence. This is consistent with the previous studies that have found a negative association between FCTC implementation and smoking prevalence [11–14]. The negative relationships between FCTC variables and smoking prevalence reiterate the importance of FCTC ratification for supporting national tobacco control efforts. However, mere ratification of FCTC should not be the main concern for the countries. It is rather the national law adopted after FCTC that matters the most. In sum, FCTC ratification is highly important as the first step and the legal basis for tobacco control measures adoption. Nevertheless, without strong law enforcement at national level, FCTC ratification may have no impact to the country’s smoking prevalence rates.

The second key finding of the study is that changes in smoking prevalence—which is also influenced by FCTC ratification—does not necessarily influence macroeconomic indicators (in this case GDP per capita). GDP per capita is a very broad macroeconomic indicator determined by numbers of factors. This study finds that cigarette consumption, portrayed by smoking prevalence, is not a determinant of a country’s GDP per capita. This finding refutes arguments that a declining tobacco industry will negatively impact the economy. The data on tobacco industry economic contribution (i.e., through tobacco taxes, labour absorption, and tobacco farming, and even tobacco exports), primarily in major tobacco producing countries such as China, India, and Brazil, appears positive. We conclude that the magnitude of the economic contribution of the tobacco industry may be overstated, and that arguments in Indonesia that increased tobacco control will have dire economic impacts is not born out in the experiences of other countries that have ratified the FCTC [15]. Furthermore, we question the value of the economic contribution of the tobacco industry in light of the associated health costs due to increasing smoking related NCDs, loss of productivity, and premature deaths [16, 17]. Negative externalities created by smoking are frequently glossed over by tobacco industry proponents. A 2015 study in the United Kingdom implies that the total direct tobacco of smoking has been approximated from £2.7 billion to £5.2 billion, that was equivalent to 5% of the total health budget [16]. In addition, a 2006 study in China, the largest tobacco producing country, found that compared to the other cash crops, tobacco has the lowest economic rate of return as government tax revenue from the tobacco industry continues to decline [18].

Intuitively, it is safe to say that FCTC ratification (leading to declining cigarette consumption) is not necessarily related to changes in GDP per capita. The findings of this study should be taken in conjunction with others that suggest that declining smoking prevalence as the result of a more comprehensive tobacco control effort under FCTC ratification can reduce a country’s healthcare costs. Previous studies have argued that smoking cessation is related to a significant reduction in healthcare costs [19, 20]. Moreover, reducing smoking prevalence can be done at relatively low cost, resulting in long-term benefits such as decreased health spending and improved labour productivity [21, 22]. In addition, raising tobacco taxes could prove mutually beneficial for the economy and health sector. Increasing tobacco tax rates will simultaneously increase government tax revenue and decrease tobacco-related medical costs [23]. This adds to yet more evidence for the importance of demand-side tobacco control mechanisms.

As important as demand-side control, supply-side tobacco control will also yield economic and health benefits. Controlling for tobacco supply, which accounted for in the FCTC, is important for the social welfare of groups impacted by increased tobacco control measures. As one of the main concerns that hinders Indonesia from ratifying the FCTC is the tobacco farmers and tobacco industry workers welfares, it is important for the government to recognise and address the potential negative impacts of FCTC measures on these groups. Nevertheless, Indonesia’s domestic tobacco industry relies heavily on imported tobacco products, so the economic impact on farmers, in particular, may be exaggerated [24]. Article 17 of the FCTC specifically mentions the need to support viable alternative economic activities for the impacted groups. If implemented in conjunction with other FCTC articles, the economic ramifications for workers associated with the tobacco industry can be minimised.

## Conclusions

Investigating the potential economic impact of FCTC is important for both academic discussion and the policymaking process. The lack of academic investigation on this topic provides space negative speculation and political rhetoric against the FCTC protocol. Investigating and drawing lessons from the experiences of other countries can help policymakers to understand that ratifying the FCTC and implementing MPOWER measures will benefit tobacco control efforts and will not necessarily have a negative impact on Indonesia’s macroeconomic indicators. Contrary to what supporters of the tobacco industry espouse, the FCTC is harmless for the economy.

Upon discussing the importance of controlling for tobacco demand and supply, FCTC ratification, which in this paper found to be one of the factors that influences the decline in smoking prevalence, will be the important key to accelerate the tobacco demand and supply control in a country. As FCTC provides comprehensive measures on tobacco control actions, FCTC ratification, which this paper found to have no direct impact on the macroeconomic indicator, will assist the country to accelerate the decrease in smoking prevalence. However, we acknowledge that we cannot differentiate the countries based on their economic share of the tobacco industry. This limitation will have subsequent consequences on our results and interpretations as our model cannot capture the exact size of the impact of FCTC’s policy measures to the economy in the short-term.

## Data Availability

Data is available upon request to: Rifai Afin (rifai.afin@trunojoyo.ac.id)

## Footnotes

1 MPOWER is WHO’s abbreviation for its six measures to assist country’s tobacco control implementation. MPOWER stands for Monitor tobacco use and prevention policies; Protect people from tobacco smoke; Offer help to quit tobacco use; Warn about the dangers of tobacco; Enforce bans on tobacco advertising, promotion and sponsorship; and Raise taxes on tobacco

## References

[1] Kesehatan K (2018). Riset Kesehatan Dasar.

[2] World Bank (2021). Prevalence of current tobacco use (% of adults). World Bank.

[3] World Bank (2021). World Bank Country and Lending Groups.

[4] World Bank. Smoking prevalence, total (ages 15+). World Bank database: The World Bank 2020.

[5] WHO. WHO report on the global tobacco epidemic country profile. Brazil: World Health Organization; 2019.

[6] WHO. WHO report on the global tobacco epidemic country profile. Bangladesh: World Health Organization; 2019.

[7] WHO. WHO report on the global tobacco epidemic country profile. Pakistan: World Health Organization; 2019.

[8] WHO. WHO report on the global tobacco epidemic country profile. Indonesia: World Health Organization; 2019.

[9] Zheng R, Marquez PV, Ahsan A (2018). Cigarette Affordability in Indonesia: 2002–2017. World Bank.

[10] Bigwanto M (2019). Tobacco industry interference undermined tobacco tax policy in Indonesia. SEATCA.

[11] Jolene Dubray RS, Chaiton M, O'Connor S, Cohen JE (2015). The effect of MPOWER on smoking prevalence. Tob Control.

[12] Anderson CL, Becher H, Winkler V (2016). Tobacco control Progress in low and middle income countries in comparison to high income countries. Int J Environ Res Public Health.

[13] Gravely S, Giovino GA, Craig L (2017). Implementation of key demand-reduction measures of the WHO framework convention on tobacco control and change in smoking prevalence in 126 countries: an association study. Lancet Public Health.

[14] Zavala-Arciniega L, Reynales-Shigematsu LM, Levy DT (2020). Smoking trends in Mexico, 2002-2016: before and after the ratification of the WHO's framework convention on tobacco control. Tob Control.

[15] Kenneth E, Warner GAF (1995). Importance of tobacco to a country's economy: an appraisal of the tobacco industry's economic argument. Tob Control.

[16] Ekpu VU, Brown AK (2015). The economic impact of smoking and of reducing smoking prevalence: review of evidence. Tob Use Insights.

[17] Pinto M, Bardach A, Palacios A, Biz A, Alcaraz A, Rodriguez B, Augustovski F, Pichon-Riviere A (2019). Burden of smoking in Brazil and potential benefit of increasing taxes on cigarettes for the economy and for reducing morbidity and mortality. Rep Public Health.

[18] Hu T-W, Mao Z, Ong M, Tong E, Tao M, Jiang H, Hammond K, Smith KR, de Beyer J, Yurekli A (2006). China at the crossroads: the economics of tobacco and health. Tob Control.

[19] Callum C, Boyle S, Sandford A (2011). Estimating the cost of smoking to the NHS in England and the impact of declining prevalence. Health Econ Policy Law.

[20] Cohen D, Barton G (1998). The cost to society of smoking cessation. Thorax.

[21] Hurley SF, Matthews JP (2008). Cost-effectiveness of the Australian National Tobacco Campaign. Tob Control.

[22] Parrott S, Godfrey C (2004). Economics of smoking cessation. BMJ.

[23] Ahmad S, Franz GA (2008). Raising taxes to reduce smoking prevalence in the US: a simulation of the anticipated health and economic impacts. Public Health.

[24] Ahsan A, Wiyono NH, Veruswati M (2020). Comparison of tobacco import and tobacco control in five countries: lessons learned for Indonesia. Glob Health.

